# Editorial: Post-translational Modifications in Plant Nuclear Signaling: Novel Insights Into Responses to Environmental Changes

**DOI:** 10.3389/fpls.2019.00104

**Published:** 2019-02-11

**Authors:** Stephane Bourque, Christian Lindermayr, Christian Mazars

**Affiliations:** ^1^UMR Agroécologie 1347 AgroSup Dijon, INRA, CNRS 6003, Université de Bourgogne Franche-Comté, Pôle Interactions Plantes-Microorganismes ERL, Dijon, France; ^2^Helmholtz Zentrum München, Deutsches Forschungszentrum für Gesundheit und Umwelt (GmbH), Neuherberg, Germany; ^3^LRSV–Université Paul Sabatier CNRS, Pôle de Biotechnologie Végétale, Castanet Tolosan, France

**Keywords:** nucleus, post-translation modification (PTM), phosphorylation, acetylation, SUMOylation

Just imagine a Plant Science professor in front of a classroom full of interested and attentive students. Imagine what their answers to this intriguing question would be: “What are, according to you, the functions ensured by the plant cell nucleus?” It would be very surprising if some of them would answer cell signaling in response to biotic and abiotic stresses or developmental processes. Most of them would probably answer according to a classical point of view: DNA replication or gene expression. Hence it is still admitted in recent publications (see for instance Fedorenko et al., 2010) that molecules smaller than 40 kDa can diffuse freely across the nuclear envelope pores. However, Pauly et al. (2000) showed by studying nuclear Ca^2+^ signaling that elevations in the extranuclear Ca^2+^ concentration do not induce an automatic increase of nuclear [Ca^2+^] as it could be expected. Hence Ca^2+^ does not freely diffuse across the nuclear envelop pores, indicating that its transport is finely regulated. Then it becomes evident that we should now consider the nucleus as a key component of cell signaling processes leading to the regulation of specific sets of genes.

The aim of this topic was to point out how nuclear post-translational modifications (PTMs) play fundamental roles in the signaling pathways initiated in response to environmental changes. Two major points were assessed. First, different papers clearly demonstrate that the nucleus is fully equipped to perform the main PTMs: (de)phosphorylation (Bigeard and Hirt; Krysan and Colcombet), (de)acetylation (Luo et al.; Fül et al.; Ramirez-Prado et al.), oxidoreduction (Martins et al.) or SUMOylation/ubiquitination (Mazur et al.; Serrano et al.). Hence the nucleus can easily integrate a complex network of second messengers including changes in Ca^2+^ concentration, reactive oxygen species or nitric oxide. Martins et al. nicely exemplified how changes in the nuclear redox status regulate fundamental processes such as cell cycle, protein transport or transcription *via* S-nitrosylation or S-glutationylation. Activation of nuclear PTMs can also be achieved by the translocation of enzymes which are both substrates and effectors of these PTMs. This is the case for mitogen-activated protein kinases (MAPKs) that in some specific contexts, translocate from the cytosol to the nucleus upon their activation by their corresponding MAPK kinases (Bigeard and Hirt). Relocation of proteins in response to or through PTMs can also be considered at the intranuclear level. Hence in response to SUMO conjugation several Arabidopsis transcription factors were shown to be re-localized in certain nuclear foci (Mazur et al.). PTMs can also affect the behavior of nuclear proteins and *in fine* their activity. Serrano et al. highlight how the ubiquitin-proteasome system contributes to the nuclear proteome plasticity. Focusing on E3-Ub-ligases, they illustrate how these enzymes attenuate the signaling pathway once the stress has ceased and how they control the homeostasis of nuclear proteins (transcription factors, immune receptors).

The second major point of this topic concerns the target proteins of these PTMs. Of course histones are a piece of choice. The review by Ramirez-Prado et al. illustrates how removing or adding marks (phosphorylation, acetylation, methylation, or ubiquitination) on specific histone lysine residues associated to defense genes (WRKYs, pathogenesis-related proteins, etc.) mostly under the control of salicylic acid or jasmonic acid/ethylene signaling pathways, will control the outcome of the plant-microorganism interaction. A second interesting aspect in this review is the illustration of how pathogens manipulate the chromatin regulatory network of the host to achieve their infection process through for example the production of toxins inhibiting histone deacetylases (HDACs), leading to plant susceptibility. PTMs on histones are also of major importance in the response to abiotic stresses. Luo et al reviewed how HDACs, by deacetylating specific lysine residues (mainly H3K9, H3K14, and H4K15) of specific genes regulate responses to salt, drought, cold or heat. However, chromatin remodeling and *in fine* regulation of gene expression is not only linked to histone modifications. Fül et al. as a perspective in this topic remind us that subunits of key chromatin remodelers (such as MED12, MED13, and MED19A) or transcription factors like Yin Yang 1 in the response to abscisic acid are also targeted and regulated by lysine deacetylases. Hence lysine (de)acetylation regulates gene expression by acting on histones, but also on the whole transcriptional machinery.

In conclusion this topic clearly illustrates the diversity of nuclear PTMs, the crosstalks between some of them, and finally their major roles in the regulation of gene expression. A first fascinating challenge is now to decipher at the molecular level the ways cells are using to translocate signals from cytoplasm to the nucleus that regulate nuclear PTMs. A second one is to go further in the characterization of their target proteins: as it is now nicely exemplified by the interplay of histone marks in epigenetics, this is the *sine qua non* condition to fully understand the power of nuclear PTMs.

## Author Contributions

All authors listed have made a substantial, direct and intellectual contribution to the work, and approved it for publication.

### Conflict of Interest Statement

The authors declare that the research was conducted in the absence of any commercial or financial relationships that could be construed as a potential conflict of interest.

## References

[B1] Fedorenko O. Yarotskyy V. Duzhyy D. Marchenko S. (2010). The large-conductance ion channels in the nuclear envelope of central neurons. Pflügers Archiv. 460, 1045–1050. 10.1007/s00424-010-0882-5 20886229

[B2] Pauly N. Knight M. R. Thuleau P. van der Luit A. H. Moreau M. Trewavas A. J. (2000). Cell signalling: control of free calcium in plant cell nuclei. Nature 405, 754–755. 10.1038/35015671 10866186

